# MicroRNAs and Chinese Medicinal Herbs: New Possibilities in Cancer Therapy

**DOI:** 10.3390/cancers7030855

**Published:** 2015-08-24

**Authors:** Ming Hong, Ning Wang, Hor Yue Tan, Sai-Wah Tsao, Yibin Feng

**Affiliations:** 1 School of Chinese Medicine, Li Ka Shing Faculty of Medicine, The University of Hong Kong, Hong Kong, China; E-Mails: hong1986@connect.hku.hk (M.H.); ckwang@hku.hk (N.W.); hoeytan@connect.hku.hk (H.Y.T.); 2 Department of Anatomy, Li KaShing Faculty of Medicine, The University of Hong Kong, Hong Kong, China; E-Mail: gswtsao@hkucc.hku.hk

**Keywords:** microRNAs, Chinese medicinal herbs, cancer therapy, exogenous microRNAs

## Abstract

In recent decades Chinese medicine has been used worldwide as a complementary and alternative medicine to treat cancer. Plenty of studies have shown that microRNAs (miRNAs) play fundamental roles in many pathological processes, including cancer, while the anti-cancer mechanisms of Chinese medicinal herbs targeting miRNAs also have been extensively explored. Our previous studies and those of others on Chinese medicinal herbs and miRNAs in various cancer models have provided a possibility of new cancer therapies, for example, up-regulating the expression of miR-23a may activate the positive regulatory network of p53 and miR-23a involved in the mechanism underlying the anti-tumor effect of berberine in hepatocellular carcinoma (HCC). In this review, we survey the role of Chinese medicinal herbal products in regulating miRNAs in cancer and the use of mediating miRNAs for cancer treatment. In addition, the controversial roles of herb-derived exogenous miRNAs in cancer treatment are also discussed. It is expected that targeting miRNAs would provide a novel therapeutic approach in cancer therapy by improving overall response and survival outcomes in cancer treatment, especially when combined with conventional therapeutics and Chinese medicinal herbal products.

## 1. Introduction

With the rapid development of the Human Genome Project, the non-coding sequence that accounts for 99% of the total human genome is attracting more and more attention by researchers. So far, in recent decades several kinds of non-coding RNAs have been discovered and identified, including Piwi-interacting RNAs (piRNAs), short interfering RNAs (siRNAs), small nucleolar RNAs (snoRNAs) and microRNAs (miRNAs) [1,2,3]. Among these non-coding RNAs, miRNAs are the most studied ones in the last years. miRNAs are highly conserved non-coding sequences generally composed of 18–24 nucleotides. They have been shown to be involved in RNA silencing and regulation of the expression of target genes by attaching to the 3′ untranslated region (3′ UTR) of target mRNAs. miRNAs are usually transcribed by RNA polymerase II from their own genes or from introns [4,5]. After transcription as long primary miRNAs, the double-stranded RNA-binding domain protein DGCR8 and RNase III-like enzyme Drosha further process the primary miRNAs to shorter hairpin loop structures containing about 70 nucleotides. Then, a protein called Pasha will bind to Drosha to form a microprocessor complex and cleave the stem-loop structure of the primary miRNAs to form a precursor miRNA (pre-miRNA). These pre-miRNAs will be exported to the cytoplasm by the nucleocytoplasmic shuttler Exportin-5 and cleaved by the RNase III enzyme Dicer into mature miRNAs [6,7,8,9]. The mature miRNA then combine with other associated proteins to form the active RNA-induced silencing complex (RISC) and regulate the target gene expression by recruiting the 3′UTR of the target mRNA to RISC. For regulating target gene expression, miRNA can either degrade the target mRNA or regulate the translation of target mRNA.

As miRNAs are involved in many physiological functions of human cells, the dysregulation of miRNAs is associated with many kinds of human disorders such as heart and nervous system diseases, obesity and cancer [10,11,12]. In particular, many kinds of miRNAs related with the initiation and progression of cancer have been identified. The oncogenic miRNAs such as miR-155, miR-17-92, miR-21, miR-221 and miR-222 have been observed to be overexpressed in various malignancies, while the tumor suppressor miRNAs such as miR-15a/miR-16-1 and let-7 are down-regulated in cancer [13,14,15]. Previous studies have proved that miRNAs can affect all six of the hallmarks of cancer cells: (1) evading growth suppressors (miR-17-92 cluster); (2) self-sufficiency in growth signals (let-7 family); (3) enabling replicative immortality (miR-372/373 cluster); (4) evasion from apoptosis (miR-34a); (5) activating invasion and metastases (miR-10b); and (6) inducing angiogenesis (miR-210) [16]. With the rapid development of genomic technologies such as microarrays, more and more cancer-related miRNAs have been discovered in various tumor types. Although whether alterations of miRNA expression is the result of tumoriogenesis or the cause of tumor formation remains a controversial issue, some basic studies and clinical trials have already demonstrated the potential prospects of miRNA-based therapeutics in cancer [17,18]. These miRNA-based therapeutics may be used directly to target tumor cells or to complement other therapies, for example, to reduce the chemoresistance of cancer cells. The chemosensitization effect of miRNAs has been evidenced by the silencing of miR-100 and miR-199b-5p in drug-resistant small lung cancer cells and ovarian cancer cells, respectively [19,20].

Traditional Chinese medicine (TCM) is one of the most widely used adjunctive therapies for cancer patients worldwide. TCM plays an important role in cancer treatment by suppressing tumor proliferation, preventing complications, improving quality of life and reducing the side effects of conventional treatments in cancer patients [21,22,23]. So far, studies on the anti-tumor effects of TCM have mainly focused on screening the effective components from Chinese medicinal herbs and elaborating their regulatory effects on tumor-related genes and proteins [24,25]. Although research on the role of Chinese medicinal herbs in regulating non-coding RNAs remains scanty, in recent years more and more researchers have begun to recognize the importance of the anti-tumor effects of Chinese medicinal herbs targeting non-coding RNAs such as miRNAs. Herein, we systematically review current studies on the relationship between miRNAs and anti-tumor TCMs and their active components, to provide a new aspect of understanding the anti-tumor mechanisms of TCMs. In addition, we discuss a novel hypothesis of gene regulation mediated by exogenous miRNAs which may provide a brand-new possibility in cancer therapy by Chinese medicinal herbs.

## 2. Current Anti-Cancer Research on Chinese Medicinal Herbal Products Targeting miRNAs

### 2.1. Induction of Apoptosis by Chinese Medicinal Herbal Products Targeting miRNAs in Cancer

Curcumin is the principal curcuminoid of turmeric, which belongs to the ginger family. Curcumin treatment could result in a >80% knockdown of miR-21 expression in prostate cancer cells, thus increasing expression of several downstream target genes, including PTEN and PDCD4, and inducing apoptosis in prostate cancer cells. The *in vivo* experiment results also confirmed that curcumin could suppress tumor growth by miR-21-mediated tumor cell apoptosis in a xenograft mice model [26].

Berberine is a famous anti-cancer herbal product from the root of *Coptis chinensis*. A recent study has proved that berberine could induce apoptosis in multiple myeloma cells by decreasing the expression of miR-99a, miR-17 and miR-106 and regulating the TP53, Erb and MAPK signaling pathways [27]. A study from the same research group found that down-regulating of miR-21 expression by berberine could also induce multiple myeloma cell apoptosis through the IL6/STAT3 pathways [28]. A Chinese research group found that camptothecin, which is isolated from the stem and bark of *Camptotheca acuminata* could induce apoptosis in cancer cells by down-regulating miR-125b and activation of the mitochondrial apoptosis pathways by increasing the expression of the Bak1, Mcl1, and p53 genes [29]. MiR-125b decreased by approximately 55% in both HeLa cells and K562 cells after camptothecin treatment compared with the control group. Further research demonstrated that miR-125b directly targeted the 3′UTR regions of these proapoptotic genes in a camptothecin-induced mitochondrial pathway.

Resveratrol is a popular natural anti-cancer agent isolated from *Polygonum cuspidatum* and some fruit skins. Recent studies have found that resveratrol could induce apoptosis by up-regulating miR-137 levels and subsequently reducing EZH2 expression in neuroblastoma. Decreasing EZH2 expression could further induce H3K27me3 down-regulation and activation of the tumor suppressor genes CLU and NGFR [30]. In another study, resveratrol induced apoptosis by suppressing miR-21 regulation of BCL-2 protein expression in pancreatic cancer cells [31]. 

Luteolin is a natural flavone from the leaves of *Terminalia chebula*. The pro-apoptosis effects of luteolin in gastric cancer cells are related to down-regulation of Bcl-2 expression by decreasing miR-34a expression. Further studies showed that anti-miR-34a oligonucleotides could block the luteolin-induced Bcl-2 suppression in gastric cancer cells [32]. 

Genistein is a kind of isoflavone which has shown activity inducing apoptosis in prostate cancer cells. MiR-1260b was down-regulated by more than 50 percent after genistein treatment in prostate cancer cells. Knocking down miR-1260b caused inhibition of two target genes, sFRP1 and Smad4. The expression of these two genes induced apoptosis and reduced survival of prostate cancer cells [33].

Matrine is one of the major bioactive ingredients isolated from *Sophora flavescens*. The pro-apoptotic effect of matrine has been identified by many studies. It was found that matrine in combination with sorafenib could induce apoptosis in HCC cells, and the mechanism study showed that inhibition of miR-21 and the subsequent increased of PTEN expression contributed to the pro-apoptosis effect of matrine and sorafenib [34].

### 2.2. Anti-Metastasis and Anti-Angiogenesis by Chinese Medicinal Herbal Products Targeting miRNAs in Cancer

The epithelial-mesenchymal transition (EMT) is a process whereby epithelial cells transform into mesenchymal type ones and become more invasive and migratable. Many studies have confirmed the relationship between EMT and cancer metastasis [35,36]. Recent research has found that the expression of several EMT-suppressive miRNAs in colorectal cancer cells was increased by curcumin treatment. The downstream target genes of these EMT-suppressive miRNAs also decreased by curcumin treatment thus further inhibited the colorectal cancer cells’ invasion and metastasis [37]. Another study on curcumin showed its inhibitory activity on the metastasis of melanoma cells through increasing miR-33b expression and concomitantly suppressing the expression of high-mobility group AT-hook 2(HMGA2) [38]. Resveratrol is also effective in preventing cancer invasion and metastasis. Studies have shown that resveratrol could suppress the osteosarcoma cell invasion and migration *in vitro* as well as lung metastasis *in vivo* by inhibiting MMP-2 activation. Further mechanistic studies showed that this MMP-2 inhibition was mediated by miR-328 up-regulation, treatment of osteosarcoma 143B and U2OS cells with resveratrol (RESV) for 6 h can up-regulate miR-328 expression by approximately 2-fold in both cell lines [39]. Isoflavones isolated from leguminous plants have shown remarkable anti-invasion and metastasis effects in pancreatic cancer cells. Isoflavone treatment could up-regulate miR-146a levels and led to suppression of several metastasis- and angiogenesis-related genes such as IRAK-1, NF-kappaB, EGFR and MTA-2. As isoflavones are nontoxic, they may emerge as new anti-cancer natural products targeting miRNAs [40]. Panax Notoginseng Saponins (PNS) is the major class of bioactive ingredients of notoginseng, a herb extensively utilized in TCM for cancer treatment. PNS treatment could decrease the expression of miR-18a in cancer cells and further down-regulate its downstream angiogenesis-related genes such as CD34 and vWF. Interestingly, the efficacy of PNS in cancer therapy is contrary to that seen in heart disease treatment in which PNS up-regulated miR-18a expression and finally induced angiogenesis [41,42]. For *in vivo* studies, one recent research report has found that resveratrol can inhibit tumor metastasis in an immunodeficient mice model. Further mechanistic studies confirmed that resveratrol can inhibit the mesenchymal-epithelial transition process by suppressing the expression of FOXC2 through regulation of miRNA-520h-mediated signal cascade in lung cancer cells [43].

### 2.3. Proliferation Inhibition by Chinese Medicinal Herbal Products Targeting miRNAs in Cancer

The anti-cancer effects of berberine and *Coptidis* *rhizoma* have been extensively elaborated in our lab. Recently, we have demonstrated that berberine could up-regulate miR-23a expression by 5-fold compared to the control group and mediate the up-regulation of the tumor suppressive genes p21/GADD45α and induce G2/M cell cycle arrest in HCC cells [44]. According to our studies, *Coptidis* *rhizome* is also effective in reducing cancer cell proliferation by targeting miRNAs. After treating HCC cells with *Coptidis* *rhizoma* aqueous extract, both miR-21 and miR-23a were obviously up-regulated. The downstream target genes of these miRNAs still need further exploration in future studies [45]. A group in Italy found that camptothecin, which inhibited the DNA enzyme topoisomerase I, could suppress the activity ofHIF-1alpha protein by up-regulating miR-17-5p and miR-155 in human cancer cells, and thus suppress the proliferation activity of cancer cells [46]. Ginsenosides isolated from the precious Chinese medicine ginseng play an important role in enhancing the immunity of patients with cancer. The anti-tumor effect in various kinds of tumors of ginsenoside Rh2 has been reported. A recent study showed that miR-491 plays a key role in inhibiting HCC proliferation by suppressing the epidermal growth factor receptor (EGFR) signaling pathway, and treatment with ginsenoside Rh2 could up-regulate miR-491 levels and directly decrease the expression of EGFR [47]. Ginsenoside Rh2 treatment of human glioma cells could lead to up-regulation of miR-128 level by 3-fold and inhibited tumor proliferation by decreasing the expression of the miR-128 downstream targeting protein E2F3a [48]. 

As an anti-cancer alkaloid agent, matrine has been used as effective treatment against breast cancer with approval by the public health authorities in China. Recent research has shown that matrine could induce cell cycle arrest at G1/S phase in human breast cancer cells. Further mechanistic studies demonstrated that matrine could increase PTEN expression by decreasing miR-21 levels which subsequently led to dephosphorylated Akt, and further induced accumulation of Bad, p27(/KIP1) and p21(/WAF1/CIP1) [49]. The Chinese herbal formula Aidi Injection has been used as an adjunct therapy for several kinds of cancers in clinical practice in China. The therapeutic efficacy of Aidi Injection has been identified by several studies [50,51,52]. A study using microarray analysis identified 55 down-regulated miRNAs while 45 miRNAs were up-regulated after treatment with Aidi Injection in breast cancer patients. Further study demonstrated that among these 45 miRNAs, miR-126 could most remarkably suppress the proliferation of breast cancer cells and 12 downstream target genes of miR-126 were predicted by PicTar and TargetScan software [53]. Epidermal growth factor receptor (EGFR) plays a crucial role in cancer cell proliferation. Besides the conventional EGFR inhibitors erlotinib and gefitinib, the herbal flavonoid luteolin showed effect in suppressing EGFR by down-regulating the Ser/Thr kinase activity of cyclin G-associated kinase (GAK) in prostate cancer cells. According to the miRNA array analysis, overexpression of miR-630by luteolin may regulate the GAK-EGFR pathway and induce cell growth arrest [54]. *Ganoderma* fruiting body has been used as a precious herbal medicine for thousands years in some Asian countries, but *Ganoderma* spore lipid was only discovered and applied in cancer adjunctive therapy several decades ago. Recent studies have demonstrated that *Ganoderma* spore lipid inhibited cell proliferation of lung adenocarcinoma in a dose-dependent manner. Mechanistic studies revealed that *Ganoderma* spore lipid could inhibit the expression of miR-21 and obviously increase the expression of PTEN and PDCD4 [55]. 

The herb mistletoe is widely used in gynecological and obstetrical diseases as well as a complementary cancer therapy in TCM. The major active component of mistletoe called mistletoe lectin-I have been shown to display significant proliferation inhibition towards colorectal cancer cells both *in vitro* and *in vivo*. miR-135a and miR-135b were down-regulated by approximately 80% after mistletoe lectin-I treatment via degradation of their precursors. Western blot analysis showed that levels of their their target gene adenomatous polyposis coli (APC) were increased, which contributed to the anti-proliferative effect displayed by mistletoe lectin-I [56,57].

### 2.4. Suppression of Cancer Stem-Like Cells by Chinese Medicinal Herbal Products Targeting miRNAs

A novel analog of curcumin has shown inhibitory effect on cancer stem-like cells in chemoresistant colon cancer cells. A mechanistic study demonstrated that this agent could down-regulate miR-21 expression with up-regulation of PTEN and inhibition of Akt phosphorylation. As the PTEN-Akt pathway plays an important role in chemoresistance in cancer stem-like cells, this finding may provide a novel therapeutic agent targeting cancer stem-like cells [58]. Hydroxycamptothecin, a derivative of camptothecin, is also effective in suppressing cancer stem-like cells. After 72 h treatment with a low dose of hydroxycamptothecin, the *in vivo* tumorigenicity of cancer stem-like MHCC97L cells obviously decreased and the proliferation and invasion ability of these cells were also suppressed. Further studies showed that miR-122 regulation may participate in the inhibition of cancer-stem like cells by hydroxycamptochecin, along with suppression of albumin, α-fetoprotein and hepatocyte nuclear factor-4 expression [59]. Another study in Taiwan found that miR-145 and miR-200 play an important role in inhibiting the stemness property of nasopharyngeal carcinoma cells after resveratrol treatment. The herbal agent resveratrol could suppress the stemness and EMT by reactivating p53 and inducing miR-200 and miR-145 expression [60]. 

Honokiol is a bioactive ingredient isolated from a number of species of *Magnolia*. Because of its special chemical properties and high oral bioavailability, honokiol has been used as a promising herbal agent for cancer treatment. A study demonstrated that honokiol could increase miR-141 expression and further down-regulate the target gene zinc finger E-box binding homeobox 2 (ZEB2) in renal cancer cells. As ZEB2 is associated with cancer stem cell properties and EMT, honokiol could inhibit metastasis as well as the stem-like properties of cancer cells at least partly through the miR-141/ZEB2 pathway [61].

## 3. Exogenous miRNAs and Chinese Medical Herbs: A Controversial Hypothesis in Cancer Treatment

In 2012, an interesting hypothesis on exogenous miRNAs was presented by Zhang *et al.* According to their studies, plant-derived miRNAs could transfer into animal’s blood and tissues from food sources. More surprisingly, their further studies showed that the plant-derived exogenous miR-168 could down-regulate the expression level of low-density lipoprotein receptor-associated protein 1 in the liver of mice [62]. Several decades ago, biological researchers believed that extracellular RNAs are unstable in peripheral blood, so here should not be any exogenous RNA in the serum or tissues of mammals. But subsequent studies confirmed that miRNAs are highly stable in peripheral blood in mammals, where they are resistant to the extreme pH and temperature in blood and insensitive to RNase activity [63,64,65]. These pioneering findings may show that some miRNAs such as viral-derived or herb-derived miRNAs may transport and function across different species [66,67]. However, so far, only a few positive results supporting this theory have been published, while several studies have demonstrated the absence of plant exogenous miRNAs in mammalian blood [68]. Moreover, the reproducibility of Zhang’s results was recently challenged by several authors. For example, Tosar *et al.*, attributed Zhang’s results to an underestimated effect of contamination [69]. These opposing ideas remind us that it is still too early to draw any conclusions about inhibition of cancer progression by herb-derived exogenous miRNAs [68,70,71]. In fact, it seems more convincing that miRNAs may act as interactive factors between heterogeneous cell types in the human body system when using Chinese herbal medicines to treat cancers. It was found that by treatment with the natural compound epigallocatechin gallate (EGCG), expression of miR-16 in breast cancer cells-derived exosome can be up-regulated, and this cancer cell-derived exosome could be absorbed by tumor associated macrophages (TAMs) and lead to functional alteration of the TAMs [72]. However, Zhang’s theory still deserves further exploration, followed by elimination of possible contamination and involvement of indirect regulation of Chinese herbal medicines on endogenous miRNAs, in this case, functional analysis of exogenous miRNAs by oral intake of miRNA-containing herbal products. This would be a new area with lots of work to do including profiling endogenous and exogenous miRNAs, identifying the endogenous targets of exogenous miRNAs and elaborating the possible mechanism(s) of heterogeneous interaction between mRNAs and miRNAs.

## 4. Conclusions

According to the previous studies, miRNAs target more than 5000 human genes, which represent probably 30% of the human genome [73]. These single-strand small non-coding RNAs are critically involved in regulating gene expression and play an important role in the pathogenesis and progression of many human diseases, including cancer. By regulating their target genes, miRNAs suppress tumoriogenesis and the progression of cancer by inducing apoptosis, arresting cell cycle, inhibiting cancer stem-like cells and suppressing metastasis or angiogenesis. Based on our original research and a literature study, 14 Chinese medical herbal products including herbal isolates, herbal extracts and herbal compounds can effectively inhibit carcinogenesis, progression and metastasis by targeting different miRNAs (Table 1). Among these miRNAs regulated by Chinese medical herbal products, miR-21 was the most reported miRNA that has been extensively studied in various cancers. PTEN and PDCD4 are the common target genes regulated by miR-21 and both contribute to the anti-cancer effect of herbal products. Another interesting observation is the regulation of miR-18a in different type of tissues by the natural agent PNS. PNS treatment could decrease the expression of miR-18a in tumor and further down-regulate CD34 and vWF that led to angiogenesis inhibition in tumors, while an opposite activity by PNS on angiogenesis was observed in heart. Besides the regulation of endogenous miRNAs by Chinese medical herbal products, the exogenous miRNAs derived from herbs also give rise to a promising hypothesis of the mechanism of action of Chinese herbal medicine for cancer therapy, but more convincing evidence must be provided to support the therapeutic efficacy by dietary intake of herbal miRNAs. 

**Table 1:** Anti-cancer Chinese medicinal herbal products targeting miRNAs.

Chinese Medicinal Herbal Products	Cell models Employed	miRNAs	Effect in Cancer Cells	Target Genes	Reference
Berberine	Human multiple myeloma cells.	miR-99a, miR-17	Induce apoptosis	TP53Erb, MAPK	[27,28]
	SKOV3 cells	miR-106, miR-21		IL6/STAT3	
	HepG2 (p53 wild type), Hep3B (p53-deficient)	miR-23a	Anti-proliferation	Nek6	[44]
Curcumin	DU145 human prostate cancer cells	miR-21	Induce apoptosis	PTEN, PDCD4	[26]
	colon cancer HCT116 and HT-29 cells		Anti-cancer stem like cells	PTEN-Akt	[58]
	HCT116 and SW480 CRC cells.	MicroRNA-33b	Anti-metastasis	HMGA2	[37,38]
Camptothecin	HeLa and HEK293 cells	miR-155, miR-17-5p	Anti-proliferation	HIF-1alpha	[46]
	HeLa cells and PC3 cells	miR-125b	Induce apoptosis	Bak1, Mcl1, p53	[29]
	MHCC97 cells	miR-122	Anti-cancer stem like cells	albumin, alpha-fetoprotein, hepatocyte nuclear factor-4	[59]
Resveratrol	Neuro-2a and SH-SY5Y cell line	miR-137,miR-21	Induce apoptosis	EZH2, BCL-2	[30,31]
	HOS, MNNG/HOS, and 143B cells	miR-328	Anti-metastasis	MMP-2	[39]
	TW01, TW06, and HONE-1 cells	miR-145, miR-200c	Anti-cancer stem like cells	P53	[60]
	CL1-5, A549, H322 cell lines	miRNA-520h	Anti-MET process	FOXC2	[43]
Ginsenoside	SMMC-7721 cells	miR-491	Anti-proliferation	EGFR	[47]
	U251, T98MG and A172 cells	miRNA-128		E2F3a.	[48]
Luteolin	BGC-823 and SGC-7901 cells	miR-34a	Induce apoptosis	Bcl-2	[32]
		miR-630	Anti-proliferation	GAK	[54]
Isoflavone	Colo357 and Panc-1 cells	miR-146a	Anti-metastasis	IRAK-1, NF-kappaB, EGFR, MTA-2.	[40]
	PC-3 and DU-145 cells	miR-1260b	Anti-proliferation and induce apoptosis	sFRP1, Smad4.	[33]
Matrine	MCF-7 cells	miR-21	Anti-proliferation	PTEN	[49]
	HepG2 and Hep3B cells		Induce apoptosis		[34]
Aidi Injection	MCF-7 cells	mir-126	Anti-proliferation	Undiscovered.	[53]
Ganoderma spore lipid	SPC-A1 cells	miR-21	Anti-proliferation	PTEN, PDCD4	
PNS	Murine lewis lung carcinoma (LLC) cell	miR-18a	Anti-angiogenesis	CD34, vWF	
Coptidisrhizoma	MHCC97-L cells	miR-21, miR-23a	Anti-proliferation	Undiscovered	
Mistletoe lectin-I	CLY and HT-29 cells	miR-135a, miR-135b	Anti-proliferation	APC	
Honokiol	A-498 cells	miR-141	Anti-cancer stem like cells and anti-metastasis	ZEB2	

In conclusion, these Chinese medical herbal products, which have been tested extensively by *in vitro* and *in vivo* studies, have shown encouraging safety profiles and significant anti-cancer effect via targeting specific miRNAs. Chinese herbal medicines and miRNAs may provide a new possibility for alternative therapies of cancer. We hope this review on Chinese herbal medicines targeting miRNAs will contribute to a novel understanding of anti-cancer Chinese herbal medicines and shed light on their future clinical applications.
